# REM Sleep and Endothermy: Potential Sites and Mechanism of a Reciprocal Interference

**DOI:** 10.3389/fphys.2017.00624

**Published:** 2017-08-24

**Authors:** Matteo Cerri, Marco Luppi, Domenico Tupone, Giovanni Zamboni, Roberto Amici

**Affiliations:** Department of Biomedical and NeuroMotor Sciences, University of Bologna Bologna, Italy

**Keywords:** REM sleep, thermoregulation, heterothermy, median preoptic nucleus, periaqueductal gray, lateral parabrachial nucleus, orexin, melanin concentrating hormone

## Abstract

Numerous data show a reciprocal interaction between REM sleep and thermoregulation. During REM sleep, the function of thermoregulation appears to be impaired; from the other hand, the tonic activation of thermogenesis, such as during cold exposure, suppresses REM sleep occurrence. Recently, both the central neural network controlling REM sleep and the central neural network controlling thermoregulation have been progressively unraveled. Thermoregulation was shown to be controlled by a central “core” circuit, responsible for the maintenance of body temperature, modulated by a set of accessory areas. REM sleep was suggested to be controlled by a group of hypothalamic neurons overlooking at the REM sleep generating circuits within the brainstem. The two networks overlap in a few areas, and in this review, we will suggest that in such overlap may reside the explanation of the reciprocal interaction between REM sleep and thermoregulation. Considering the peculiar modulation of thermoregulation by REM sleep the result of their coincidental evolution, REM sleep may therefore be seen as a period of transient heterothermy.

The primary function of rapid-eye movement sleep (REMS) is still unknown, but the finding that the daily amount of REMS is “homeostatically” regulated (Cerri et al., 2005; Amici et al., 2008) suggests that it may satisfy some primary physiological needs. Pioneering studies showed that REMS occurrence is depressed at an ambient temperature (Ta) outside the thermoneutral range of the species (Parmeggiani and Rabini, 1967a), and thermoregulatory responses, such as shivering and panting, are suppressed during REMS (Parmeggiani and Rabini, 1967b). This thermoregulatory impairment has been confirmed by different studies (Parmeggiani, 2003; Heller, 2005). Also, since the direct warming and cooling of the preoptic area (POA) was shown to be inefficient in eliciting appropriate thermoregulatory responses during REMS, such an impairment was ascribed to a suspension in the central control of body temperature (Tb) (Parmeggiani et al., 1973, 1977; Glotzbach and Heller, 1976; Martelli et al., 2014).

However, the few studies on changes in POA neuronal thermosensitivity during sleep (Parmeggiani et al., 1983, 1986, 1987; Glotzbach and Heller, 1984; Alam et al., 1995) have not clarified the mechanisms of POA unresponsiveness during REMS. Consequently, investigations into the relationship between REMS and thermoregulation have been mostly phenomenal. A more mechanistic milieu has arisen from recent studies on thermoregulatory circuits (Morrison and Nakamura, 2011), and the critical role of the hypothalamus in sleep has been recognized (Saper et al., 2005). In this mini-review we will: (i) provide a brief data overview describing the interaction between REMS and thermoregulation; (ii) summarize the central networks regulating REMS and Tb and the areas in which they overlap and, (iii) suggest possible mechanisms of the reciprocal interaction between REMS and thermoregulation.

## REM sleep and thermoregulation

Initial studies on the interaction between sleep and thermoregulation were carried out, in different species, at both low and high Tas (Parmeggiani and Rabini, 1970; Schmidek et al., 1972; Haskell et al., 1981; Sichieri and Schmidek, 1984). They showed that REMS amount plotted against Ta values took the shape of an inverted U curve, with a maximum value moving in accordance with acclimation to Ta. In rats, the peak of REMS occurrence defined a thermoneutral zone (TNZ) that was narrower than that delimited by the minimal O_2_ consumption (Szymusiak and Satinoff, 1981). Thus, REMS occurrence is influenced by thermoregulation and declines at Tas beyond the TNZ limits. In accordance with this, not only REMS expression is higher at the circadian nadir of Tb, but also the two rhythms are phase-locked in free-running conditions (Lee et al., 2009).

It is worth noting that, in the latter condition, REMS occurrence is preceded, during Wake and NREM sleep (NREMS), by postural adjustments that optimize thermal exchanges (Parmeggiani, 1980); the potential inhibition of REMS occurrence according to Ta belongs to the same repertoire of behavioral thermoregulation. In the rat, the efficacy of this mechanism is revealed by the observation that, during the acclimation to Tas close to the TNZ boundaries, REMS occurrence is initially reduced and then restored to control levels in about 1 week (Mahapatra et al., 2005; Kaushik et al., 2012).

Since endothermic homeotherms evolved with a Tb that was much closer to the upper than to the lower limit of their lethal core temperature, the interaction between REMS and thermoregulation has mostly been addressed within the wider span of cold defense mechanisms. In the rat, this approach showed that REMS is reduced proportionally to Ta and that the REMS debt is fully recovered, following the return to TNZ, through a mechanism based on the frequency rather than the duration of episodes (Cerri et al., 2005; Amici et al., 2008). This pattern, qualitatively described in early reports (Schmidek et al., 1972; Sichieri and Schmidek, 1984), appears to conform to the energetic constrains of polyphasic sleep in small mammals (Capellini et al., 2008).

Long-term selective REMS deprivation studies have been performed in the rat (Rechtschaffen et al., 1983). The results showed that animals progressively developed a severe hypothermia, caused by an increase in heat loss (Bergmann et al., 1989). This appeared to be counteracted by behavioral thermoregulation, since deprived animals were able to select progressively higher Tas in a thermal gradient (Prete et al., 1991), but not by an increase in metabolic rate, which was concomitant with an incremental hyperphagia. These results were further clarified by the finding, in REMS-deprived rats, of an increased expression of the uncoupling protein-1 in the brown adipose tissue (BAT) and a decrease in leptin secretion (Koban and Swinson, 2005). Thus, it appears that a long-lasting deficiency of periods of central thermoregulatory unresponsiveness, represented by REMS, will progress to a malfunctioning of the different thermoeffector loops balancing Tb (Romanovsky, 2007).

The onset of REMS is characterized by an increase in hypothalamic temperature (Thy) (Kawamura and Sawyer, 1965), which is usually in the range of decimals of a degree and evident even outside the TNZ (Parmeggiani, 2003). This change was conditionally coupled to the increase in cerebral blood flow characterizing REMS (Franzini, 1992) until it was shown that it mainly depends on an larger increase in the flow from vertebral arteries compared to that from carotid arteries, the former circle supplying the brain with warmer blood than the latter (Azzaroni and Parmeggiani, 1993).

The thermal irrelevance of the Thy increase during REMS episodes contrasts with its strictly controlled decrease, during NREMS episodes leading to REMS occurrence (Parmeggiani et al., 1975). With respect to this, a quantitative study on the slope of that decrease showed the possibility to predict the onset of REMS within a 1 min interval (Capitani et al., 2005).

The thermal irresponsiveness of POA, decrease in the overall O_2_ consumption and increase in the overall heat loss (Roussel and Bittel, 1979; Schmidek et al., 1983), probably due to changes in peripheral vasomotion in opposition to a homeothermic control of Tb (Parmeggiani et al., 1977; Franzini et al., 1982; Alfoldi et al., 1990), support the view that REMS is a poikilothermic state, while Wake and NREMS remain homeothermic (Parmeggiani, 2003).

By taking into account the autonomic irregularities associated with REMS (Parmeggiani, 1980; Amici et al., 2014) this dichotomy may be extended to systemic physiological regulations, indicating a poikilostatic control for REMS and the permanence of a homeostatic control for Wake and NREMS (Parmeggiani, 2003). According to this view, POA thermal irresponsiveness depends on an impairment of diencephalic integrative activity. Thus, physiological regulation during REMS should mainly operate through a brainstem reflex activity, destitute of the hypothalamic control (Parmeggiani, 2003). However, hypothalamic osmoregulation, which is phylogenetically older than thermoregulation, is not impaired during REMS (Luppi et al., 2010), and REMS occurrence is hardly affected by a long-lasting water deprivation (Martelli et al., 2012). These results raise the possibility that the distinctive trait of REMS is the development of a poikilothermic condition, and this may be the reason why REMS occurrence is so intensely influenced by thermoregulation.

## The central circuits controlling REM sleep and thermoregulation

### The central network controlling REM sleep

The neural network controlling REMS onset was initially outlined in the cat (Jouvet, 1962) and, later, in the rat (Luppi et al., 2014, 2017). In the cat, a central role in REMS generation has been attributed to pontine cholinoceptive/cholinergic neurons (Vanni-Mercier et al., 1989; Sakai and Koyama, 1996). In the rat, the crucial role of pontine structures in REMS generation has been confirmed, and general agreement has been reached regarding the prominent role of REMS-on glutamatergic neurons of the sublaterodorsal tegmental nucleus (SLD) (Luppi et al., 2014, 2017). Projections from SLD have been shown to activate neural networks underlying both brain cortical and somatic hallmarks of REMS (Luppi et al., 2014, 2017).

SLD neurons receive a tonic excitatory glutamatergic input from different brain areas and are kept inhibited during Wake and NREMS by projections from REMS-off neurons of the ventrolateral periaqueductal gray (VlPAG) and the dorsal deep mesencephalic reticular nuclei (dDPMe) (Luppi et al., 2014, 2017). VlPAG/dDPMe REMS-off neurons are excited by both orexin neurons in the lateral hypothalamus (LH) and monoaminergic neurons in the brainstem and tuberomammillary wake-promoting areas.

The inhibition of these VlPAG/dDPMe REMS-off neurons is apparently crucial for REMS onset. Active inhibition is promoted by a sub-population of VlPAG GABAergic REMS-on neurons, while disfacilitation is due to the suppression of firing, during REMS, of monoaminergic wake-promoting neurons, to which GABAergic REMS-on VlPAG neurons also send their terminals. It has been proposed that further inhibitory inputs arise from ascending GABAergic projections from the medulla in both rats (Luppi et al., 2014, 2017) and mice (Weber et al., 2015).

A crucial role in the inhibition of VlPAG/dDPMe REMS-off neurons is played by REMS-on neurons of the posterior hypothalamus, including LH, zona incerta, and perifornical hypothalamus, many of which release GABAand/or the peptide melanin-concentrating hormone (MCH) (Luppi et al., 2014, 2017). In fact, this group of neurons is considered the “master generator” of REMS (Luppi et al., 2014). The central role of the hypothalamic MCH/GABAergic neurons in REMS occurrence has been underlined by optogenetic and chemogenetic studies in rats (Jego et al., 2013) and mice (Vetrivelan et al., 2016), respectively. MCH neurons, inhibited by monoaminergic wake-promoting neurons, may also contribute to the active inhibition of orexin neurons in the LH during REMS.

At a preoptic-hypothalamic level, the median preoptic nucleus (MnPO) and the ventrolateral preoptic nucleus (VLPO) play a role in REMS regulation (Gvilia et al., 2006; Dentico et al., 2009). In both structures, the degree of cellular activity appears to be related to the homeostatic need for REMS, which, increases during REMS deprivation and decreases during the following REMS rebound. It has been suggested that both structures are part of the network for the switching-off of the brainstem and hypothalamic wake-promoting centers when sleep need is increased, but the MnPO has been shown to have a closer link with REMS regulation (Szymusiak and McGinty, 2008; McKinley et al., 2015). A similar REMS-related pattern has been found at the pontine level in the Lateral Parabrachial Nucleus (lPBN), largely active during both REMS deprivation and the following REMS rebound (Verret et al., 2005).

### The central network controlling thermoregulation

Research in thermoregulation has led to a better definition of the neural pathways through which cutaneous thermal receptors activate BAT thermogenesis, as well as shivering thermogenesis, and cutaneous vasoconstriction (CVC) for heat retention, necessary for cold defense (Cano et al., 2003; Nakamura and Morrison, 2007, 2008, 2010, 2011; Morrison and Nakamura, 2011; Morrison et al., 2012).

Cold and warm signals from the skin are transmitted, through glutamatergic second order ascending neurons from the dorsal horn to the externolateral- (el) and dorsolateral- (dl) PBN neurons, respectively. From here, elPBN glutamatergic neurons convey the cold thermal signal to the GABAergic Median preoptic (MnPO) neurons (Tan et al., 2016), which in turn inhibit the warm-sensitive GABAergic neurons within the medial preoptic (MPO) projecting to the dorso-medial hypothalamus (DMH) and raphe pallidus (RPa). This leads to an increased activity of thermogenesis-promoting neurons in the DMH, which provide the main excitatory drive for the rostral RPa (rRPa) premotor neurons with consequent activation of thermogenesis (Morrison et al., 1999; Cerri et al., 2010). Alternatively, warm thermal signals retransmitted by dlPBN glutamatergic neurons activate the MnPO glutamatergic neurons, which in turn activate MPO GABAergic neurons projecting to the DMH and rRPa. This leads to an inhibition of thermogenesis-promoting neurons in the DMH, reducing the excitatory drive to the rRPa premotor neurons. The inhibition of RPa neurons increases thermal dissipation and leads to a reduction in body temperature (Cerri et al., 2010, 2013).

The thermoregulatory network sends its branches to several brain areas that control metabolic, cardiovascular, osmolar and respiratory functions and, conversely, receives feedback from these areas, thus modulating thermoregulatory responses (Morrison et al., 2014). Among these areas, the role of the LH and the PAG is of particular interest in the context of this review.

Two relevant populations of neurons that modulate thermoregulation are located within the LH: orexin neurons and MCH neurons. Orexin neurons send direct projections to the rRPa (Oldfield et al., 2002; Berthoud et al., 2005; Tupone et al., 2011), are directly involved in the modulation of BAT thermogenesis (Tupone et al., 2011; Luong and Carrive, 2012), are indispensable to mediate the prostaglandin E2-induced fever, and are necessary for the defense against environmental cooling in mice (Takahashi et al., 2013). MCH signal deficiency has been shown to increase Tb (Ahnaou et al., 2011; Takase et al., 2014).

PAG neurons receive projections from the main thermoregulatory hypothalamic nuclei (Rizvi et al., 1992; Yoshida et al., 2005) and project directly to the rRPa (Hermann et al., 1997) and, multi-synaptically, to BAT (Cano et al., 2003), mostly from the ventromedial and the ventrolateral regions, respectively. The caudal portion of the lateral PAG contains BAT sympatho-excitatory neurons (Chen et al., 2002; Nakamura and Morrison, 2007), whereas the rostral PAG contains BAT sympatho-inhibitory neurons (Rathner and Morrison, 2006).

## Potential sites and mechanisms at the base of the interaction between REM sleep and thermoregulation

**Figure 1:**
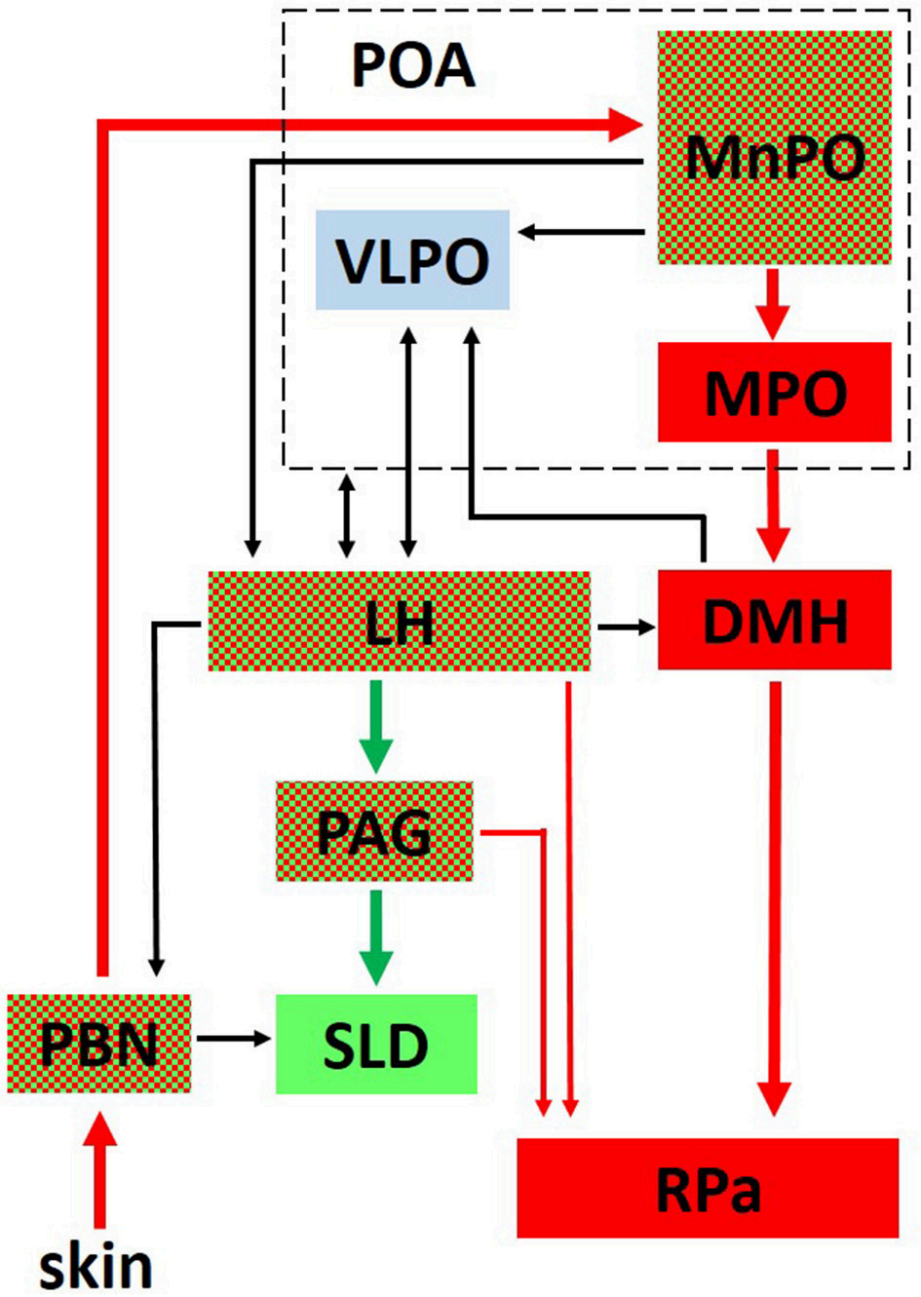
This figure outlines the interaction between the central network controlling thermoregulation (in red) and the central network controlling REM sleep (in green). Areas belonging to both networks are red and green checked. Interactions of both networks with the ventrolateral preoptic nucleus (VLPO, in light blue), a critical part of the network controlling non-REM sleep onset, are also shown. PBN, Parabrachial Nucleus; MPO, Medial Preoptic; MnPO, Median Preoptic; DMH, Dorsomedial Hypothalamus; RPa, Raphe Pallidus; LH, Lateral Hypothalamus, PAG, Periaqueductal Gray; SLD, sublaterodorsal tegmental nucleus; Thin lines represent a modulatory influence.

The tight reciprocal link between REMS and thermoregulation suggests the existence of mechanisms underlying this interaction at the level of the brain areas shared by the two regulatory networks. In fact, it is noteworthy that the sleep network and the thermoregulation network overlap in some brain areas (Figure 1). This overlap is particularly evident in the case of the LH, where two populations of wake-promoting neurons, expressing orexin (Adamantidis et al., 2007) or GABA (Venner et al., 2016), are intermingled with a population of REMS-promoting neurons expressing GABA and MCH (Hanriot et al., 2007). MCH neurons are also segregated from the orexin neurons (Kerman et al., 2007), and send reciprocal connections to each other (Guan et al., 2002). The entire area also has relevant effects on thermoregulation and behavioral state regulation when activated (Cerri and Morrison, 2005; Di Cristoforo et al., 2015), or inhibited (Cerri et al., 2014). In particular, the LH inhibition by the local delivery of the GABA-A agonist muscimol led to REMS suppression in rats (Clement et al., 2012; Cerri et al., 2014).

Another overlap between the two networks occurs at the POA level, in particular, the MnPO. The MnPO is a very important integrative site for homeostatic function, since it receives inputs from different sensory pathways and contains osmoresponsive, thermoresponsive, and sleep-related neurons, which, to some extent, reciprocally interact (McKinley et al., 2015). Intrinsic MnPO GABAergic neurons, which are activated by projections from the elPBN might directly or indirectly inhibit the REMS-related neurons in the MnPO, contributing to Wake enhancement and REMS suppression at a low Ta.

A further possible site of overlap between REMS regulation and thermoregulation is the VlPAG. On one hand, a consistent number of either REMS-off or REMS-on neurons have been found in VlPAG (Sapin et al., 2009). On the other hand, neurons from this region directly project to the RPa (Hermann et al., 1997; Cano et al., 2003), some of which are able to indirectly promote BAT activity (Chen et al., 2002; Nakamura and Morrison, 2007). However, these neurons appear to be differently controlled. In fact, while REMS-off neurons are apparently kept active by orexinergic and monoaminergic afferents (Luppi et al., 2014, 2017), thermoregulatory neurons apparently receive inputs from the DMH/dorsal hypothalamic area (Yoshida et al., 2005) and the MPO (Rizvi et al., 1992). As discussed by others (Martelli et al., 2013), a further possible site of overlap can be found at the level of the lPBN, since lPBN neurons may influence REMS occurrence via direct projections to the SLD (Boissard et al., 2003).

## Conclusions

A way to consider changes in the activity of MnPO in REMS deprivation and recovery (Gvilia et al., 2006; Dentico et al., 2009) is that this nucleus belongs to a preoptic set which is thought to form, with the DMH, a visceromotor pattern generator (HVPG) (Thompson and Swanson, 2003). As suggested by the normality of fluid regulation (Luppi et al., 2010; Martelli et al., 2012), the thermal irresponsiveness of POA may change the visceromotor response patterns of HPVG.

The clamping of Thy, during REMS, by a diathermic warming of the thermally irresponsive POA, doubled episode duration even at a Ta well below the lower limit of TNZ (Parmeggiani et al., 1974), and this extra REMS was fully accounted for within deprivation-recovery processes (Parmeggiani et al., 1980). This increase in REMS duration may be interpreted as a direct thermal effect on sleep-regulating circuits, whereas hypothermia has the opposite effect (Jones et al., 2008; Del Vecchio et al., 2014). However, its striking efficacy may, alternatively, be viewed as a sign that Thy is monitored by POA before REMS onset, and by the DMH subdivision of HPVG during its occurrence. Along these lines, the diathermic warming of POA did not change c-FOS expression in that area, but suppressed a c-FOS increase induced in DMH by previous cold exposure (Yoshida et al., 2002). The potential role for DMH in the peculiar thermoregulatory set of REMS is further supported by the finding that a transection separating POA from DMH transforms the input of peripheral thermoreceptors into a response, by thermal effectors, that is directly proportional to Ta (inverted thermoregulation) (Tupone et al., 2017).

Thus, taken together, these results suggest that REMS may be considered as a transient heterothermic state fulfilling, within the far-reaching protection of a rest period, specific needs of endotherms brain activity, rather than energy saving. This view appears in line with the hypothesis of a coevolution of REMS and thermoregulation (Lee Kavanau, 2002) and the observation of an occurrence of REMS-like episodes in hibernating lemurs only at the highest Ta still compatible with torpor (Krystal et al., 2013; Blanco et al., 2016).

On these bases, the interplay between REMS and thermoregulation may be linked to the simultaneous evolution of the two functions, and the sharing of regulatory areas may be the results of some evolutionary constraint in terms of developmental physiology. Thus, the study of the interaction between REMS and thermoregulation may open new perspectives on how the two functions developed and shed light on the yet unknown purpose of REMS.

## Author contributions

This manuscript is the result of the common effert of MC, ML, DT, GZ, and RA. All the authors contributed to the development of the manuscript.

### Conflict of interest statement

The authors declare that the research was conducted in the absence of any commercial or financial relationships that could be construed as a potential conflict of interest.
